# Prognostic factors and outcome of Liposarcoma patients: a retrospective evaluation over 15 years

**DOI:** 10.1186/s12885-017-3398-y

**Published:** 2017-06-12

**Authors:** Carolin Knebel, Ulrich Lenze, Florian Pohlig, Florian Lenze, Norbert Harrasser, Christian Suren, Jonathan Breitenbach, Hans Rechl, Rüdiger von Eisenhart-Rothe, Heinrich M. L. Mühlhofer

**Affiliations:** Department of Orthopedics and Sports Orthopedics, Technical University of Munich, Ismaninger Str. 22, 81675 Munich, Germany

**Keywords:** Liposarcoma, Prognostic factors, Outcome, Survival

## Abstract

**Background:**

Soft tissue sarcomas are rare entities with over 50 histological subtypes. Liposarcoma (LS) is the most common neoplasm in this group; it is a complex neoplasm that is divided into different histological subtypes. Different therapy options, such as surgical resection, radiation, and chemotherapy, are available. Depending on the subtype, location, status of the resection margins and metastatic status, different therapy options are used. Therefore, the aim of this study was to determine the prognostic factors influencing the survival of patients affected by LS with consideration for the grading, histological subtype, state of the resection margin, size, location, metastases and local recurrence in a retrospective, single-centre analysis over 15 years.

**Methods:**

We included 133 patients (male/female = 67/66) in this study. We recorded the histologic subtype, grade, TNM classification, localization, biopsy technique, tumour margins, number of operations, complications, radiation and dose, chemotherapy, survival, recrudescence, metastases and follow-up. Survivorship analysis was performed.

**Results:**

We detected 56 (43%; 95%-CI 34.6–51.6%) atypical LS cases, 21 (16.2%; 95%-CI 9.8–22.5) dedifferentiated LS cases, 40 (30.8%; 95%-CI 22.8–38.7) myxoid LS cases and 12 (9.2%; 95%-CI 4.3–14.2) pleomorphic LS cases. G1 was the most common grade, which was followed by G3. Negative margins (R0) were detected in 67 cases (53.6%; 95%-CI 44.9–62.3) after surgical resection. Local recurrence was detected in 23.6% of cases. The presence of metastases and dedifferentiated LS subtype as well as negative margins, grade and tumour size are significant prognostic factors of the survival rates (*p* < 0.015).

**Conclusion:**

Grading, LS subtype, negative margins after surgery, metastases and tumour size are independently associated with disease-specific survival, and patients with local recurrence had lower survival rates. We hope our investigation may facilitate a further prospective study and clinical decision-making in LS.

## Background

Soft tissue sarcomas are rare entities with over 50 histological subtypes. The annual incidence of soft tissue sarcomas is slightly higher than bone sarcoma, but at approximately 2–5 per 100,000 per year, it remains a rare disease [1, 2]. In the U.S., nearly 5000 patients die from this disease per year; however, approximately 12,000 patients will be diagnosed annually [2]. Treatments for soft tissue sarcomas depend on the histological subtype and vary from primary surgical resection to (neo-)adjuvant radiation and/or (neo-)adjuvant cytotoxic chemotherapy with different agents. Diagnostic assessment includes the clinical history and examination, proper imaging and tissue biopsy depicting the crucial step in the diagnostic cascade to establish a histological diagnosis for the therapeutic strategy [3, 4]. Among the wide variety of soft tissue sarcomas, liposarcoma (LS) is the most common neoplasm in this group. The WHO describes LS as a complex neoplasm as well as a heterogeneous group of different subtypes, such as atypical lipomatous tumour, well-differentiated LS, dedifferentiated LS, myxoid LS and pleomorphic LS [5]. However, depending on the subtype, different treatment strategies are used. Surgical resection with histologically negative margins is the gold standard in oncologic therapy; however, depending on the subtype, the location, status of resection and metastases, other therapy options are used. Few studies evaluating these determining factors of LS consider the histological subtypes [6–10].

Therefore, the aim of this study was to determine the prognostic factors influencing the survival of patients affected by LS with consideration for the grading, histological subtype, state of resection, size, location, metastases and recrudescence in a retrospective, single-centre analysis over 15 years.

## Methods

The approval of the institutional review board and written consent from each subject prior to inclusion were obtained before initiating the study (Ethikkommission der Medizinischen Fakultät, Technische Universität München). We retrospectively reviewed our institution’s database for patients who underwent treatment for the diagnosis of LS from October 1997 to November 2012. We identified 133 patients (male/female = 67/66) with a median age of 55.1 years (14–86 years) at the time of LS diagnosis. The distribution of subtypes included in this study is illustrated in Table 1. Of the 133 patients, 130 (130 tumours) were included in the present study (3 patients with 3 tumours were excluded due to the lack of a clear histological definition). A cohort of 101 was primarily treated at our institution; 32 had at least one operation at another hospital, and 2 patients had developed metastases at the time of diagnosis. We recorded the age (diagnosis), gender (male/female), histologic subtype (atypical/well-differentiated LS, dedifferentiated LS, myxoid LS and pleomorphic LS), grading (G1, G2, and G3), TNM classification, localization, biopsy technique, tumour margins (R0: negative/clean margins; R1: positive/involved margins (microscopic); R2: positive/involved margins (macroscopic), Rx: the presence of residual tumour cannot be assessed), number of operations, complications, radiation (adjuvant, neoadjuvant, and intraoperative) and dose, chemotherapy, survival, recrudescence, metastases (time of occurrence/localization), and follow-up (months from operation). Survivorship analysis was performed using the Kaplan-Meier survivorship method. Prognostic factors and their influence on the mortality were determined with the log-rank test. All data are reported as the mean, standard deviation, and percentage, where applicable. Statistical analysis was performed using SPSS 2.0 (IBM, Armonk, NY, USA).

**Table 1:** Histological subtype

Histological subtype	Number of patients
Atypical lipomatous tumour	56
Dedifferentiated liposarcoma	21
Myxoid liposarcoma	40
Pleomorphic liposarcoma	12
Mixed-type liposarcoma	1
	Total: 130 patients

## Results

Overall, we detected 56 (43%; 95%-CI 34.6–51.6%) atypical LS cases, 21 (16.2%; 95%-CI 9.8–22.5) dedifferentiated LS cases, 40 (30.8%; 95%-CI 22.8–38.7) myxoid LS cases, 12 (9.2%; 95%-CI 4.3–14.2) pleomorphic LS cases, and 1 (0.8%; 95%-CI 0–2.3) mixed-type (blending of atypical LS and myxoid LS) LS case. Concerning the tumour grade, we determined that there were 80 (60.6%; 95%-CI 52.3–68.9) G1 tumours, 19 (14.4%; 95%-CI 8.4–20.4) G2 tumours and 33 (25%; 95%-CI 17.6–32.4) G3 tumours in our cohort; one tumour had to be excluded because exact grading could not be classified in histology. With respect to the tumour size, we observed 5 (4.3%; 95%-CI 0.6–8.0) tumours that were smaller than 5 cm and 111 (95.7%; 95%-CI 92.0–99.4) cases larger than 5 cm. Seventeen cases were excluded because the tumour size could not be retrospectively evaluated. The TNM Classification of all cases and TNM Classification divided by histological subtype is provided in Tables 2 and 3. We demonstrated an accumulation of LS at the lower limb with 72.9% (95%-CI 65.4–80.5) of the cases and especially at the thigh in 67.7% (95%-CI 59.7–75.6) of all tumours. Localization of the different histological subtypes and the confidence interval are shown in Table 4.

**Table 2:** TNM-classification (2002)

	Frequency	Percent	Valid percent	Cumulative percent
Valid pT1a, pNx, pMx	3	2,3	2,3	2,3
pT1b, pNx, pMx	2	1,5	1,5	3,8
pT2a, pNx, pMx	10	7,5	7,5	11,3
pT2b, pNx, pMx	82	61,7	61,7	72,9
pT2b, pNx, cM1	2	1,5	1,5	74,4
no TNM	7	5,3	5,3	79,7
pT2, pNx, pMx	1	0,8	0,8	80,5
rpT2b, Nx, Mx	5	3,8	3,8	84,2
ypT2b, pNx, pMx	11	8,3	8,3	92,5
subfascial	10	7,5	7,5	100,0
Total	133	100,0	100,0

**Table 3:** Crosstabulation: TNM-classification/tumor entity

		Tumor entity					Total
		ALT	DLS	MLS	PLS	LSM	
TNM-classification	pT1a, pNx, pMx	1	0	0	2	0	3
	pT1b, pNx, pMx	0	2	0	0	0	2
	pT2a, pNx, pMx	5	0	4	1	0	10
	pT2b, pNx, pMx	46	8	20	5	1	80
	pT2b, pNx, cM1	0	1	1	0	0	2
	kein TNM vorhanden	1	2	3	1	0	7
	pT2, pNx, pMx	0	0	1	0	0	1
	rpT2b, Nx, Mx	0	1	4	0	0	5
	ypT2b, pNx, pMx	1	5	4	1	0	11
	subfascial	2	2	3	2	0	9
Total		56	21	40	12	1	130

*ALT* Atypical lipomatous tumour, *DLS* Dedifferentiated liposarcoma, *MLS* Myxoid liposarcoma, *PLS* Pleomorphic liposarcoma, *LSM* Mixed-type liposarcoma

**Table 4:** Localisation of tumors

Localisation of tumors	Percent of patients
Lower extremity	72,93%
Upper extremity	11,28%
Pelvic	6,02%
Retroperitoneum	3,76%
Dorsum	3,01%
Head and Neck	0,75%
Thorax wall	0,75%
Spermatic cord	0,75%
Multilocular	0,75%
	Total 100%

Surgical resection was performed in 128 patients (96.2%; 95%-CI 93–95); 5 patients could not undergo an operation because of tumour proliferation. Negative margins (R0) were detected in 67 cases (53.6%; 95%-CI 44.9–62.3); 44 (35.2%; 95%-CI 26.8–43.6) cases were classified R1, 12 cases as R2 (9.6%; 95%-CI 4.4–14.8), 2 cases could not be verified, and 3 cases had no available information about the margins. In the R1 group, a second operation was performed in 7 cases (15.9%. 95%-CI 5.1–26.7). The relationship between the histological subtype and margins is provided in Table 5.

**Table 5:** Tumor entity/resection margins

		Resection margins				Total
		R0	R1	R2	Rx	
Tumor entity	Atypical lipomatous tumour	30	22	3	0	55
	Dedifferentiated liposarcoma	7	9	4	0	20
	Myxoid liposarcoma	22	10	4	1	37
	Pleomorphic liposarcoma	7	2	0	1	10
Total		66	43	11	2	122

Fifty-five patients (43.0%, 95%-CI 34.4–51.0%) underwent radiation, and 18 patients (14.1%, 95%-CI 8.0–20.1) underwent chemotherapy. Details are provided in Tables 6 and 7. Local recurrence was detected in 29 cases (23.6%; 95%-CI 16.1–31.1). Patients with a high-grade LS G3 were more likely to suffer from local recurrence (45.5%; 95%-CI 28.5–62.4) than G1 LS (12.5%; 95%-CI 5.3–19.7) and G2 (15.8%; 95%-CI 0–31%). The rates of local recurrence for different histological subtypes are given in Table 8.

**Table 6:** Crosstabulation radiation/tumor entity

		Tumor entity				Total
		ALT	DLS	MLS	PLS	
Radiation	preoperatively	2	3	4	1	10
	postoperatively	2	7	10	7	26
	preoperatively + intraoperatively	0	0	2	0	2
	postoperatively + intraoperatively	5	6	3	0	14
Total		9	16	19	8	52

*ALT* Atypical lipomatous tumour, *DLS* Dedifferentiated liposarcoma, *MLS* Myxoid liposarcoma, *PLS* Pleomorphic liposarcoma

**Table 7:** Crosstabulation chemotherapy/tumor entity

			Tumor entity					Total
			ALT	DLS	MLS	PLS	LSM	
Chemotherapy	Yes	Number of patients	0	5	6	6	0	17
		% of chemotherapy	0,0%	29,4%	35,3%	35,3%	0,0%	100,0%
	No	Number of patients	56	16	32	6	1	111
		% of chemotherapy	50,5%	14,4%	28,8%	5,4%	0,9%	100,0%
Total		Number of patients	56	21	38	12	1	128
		% of chemotherapy	43,8%	16,4%	29,7%	9,4%	0,8%	100,0%

*ALT* Atypical lipomatous tumour, *DLS* Dedifferentiated liposarcoma, *MLS* Myxoid liposarcoma, *PLS* Pleomorphic liposarcoma, *LSM* Mixed-type liposarcoma

**Table 8:** Local recurrence (LR) and tumor entity

			Tumor entity					Total
			ALT	DLS	MLS	PLS	LSM	
LR yes/no	Yes	Number of patients	6	10	8	4	0	28
		% of Tumor Entity	11,5%	47,6%	21,6%	36,4%	0,0%	23,0%
	No	Number of patients	46	11	29	7	1	94
		% of Tumor Entity	88,5%	52,4%	78,4%	63,6%	100,0%	77,0%
Total		Number of patients	52	21	37	11	1	122
		% of Tumor Entity	100,0%	100,0%	100,0%	100,0%	100,0%	100,0%

**Fig. 1:**
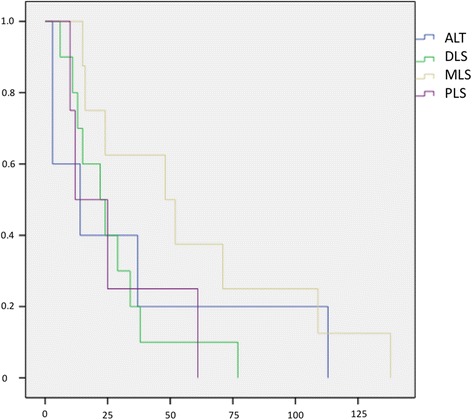
Kaplan-Meier Curve showing statistical analysis (x-axis: months, y-axis: probability) of time to first local recurrence in months after initial diagnosis of different types of liposarcoma; *p*-value: *p* < 0.05. *blue line*: ALT (atypical lipomatous tumour), *green line*: DLS (dedifferentiated liposarcoma), *yellow line*: MLS (myxoid liposarcoma), *purple line*: PLS (pleomorphic liposarcoma)

**Fig. 2:**
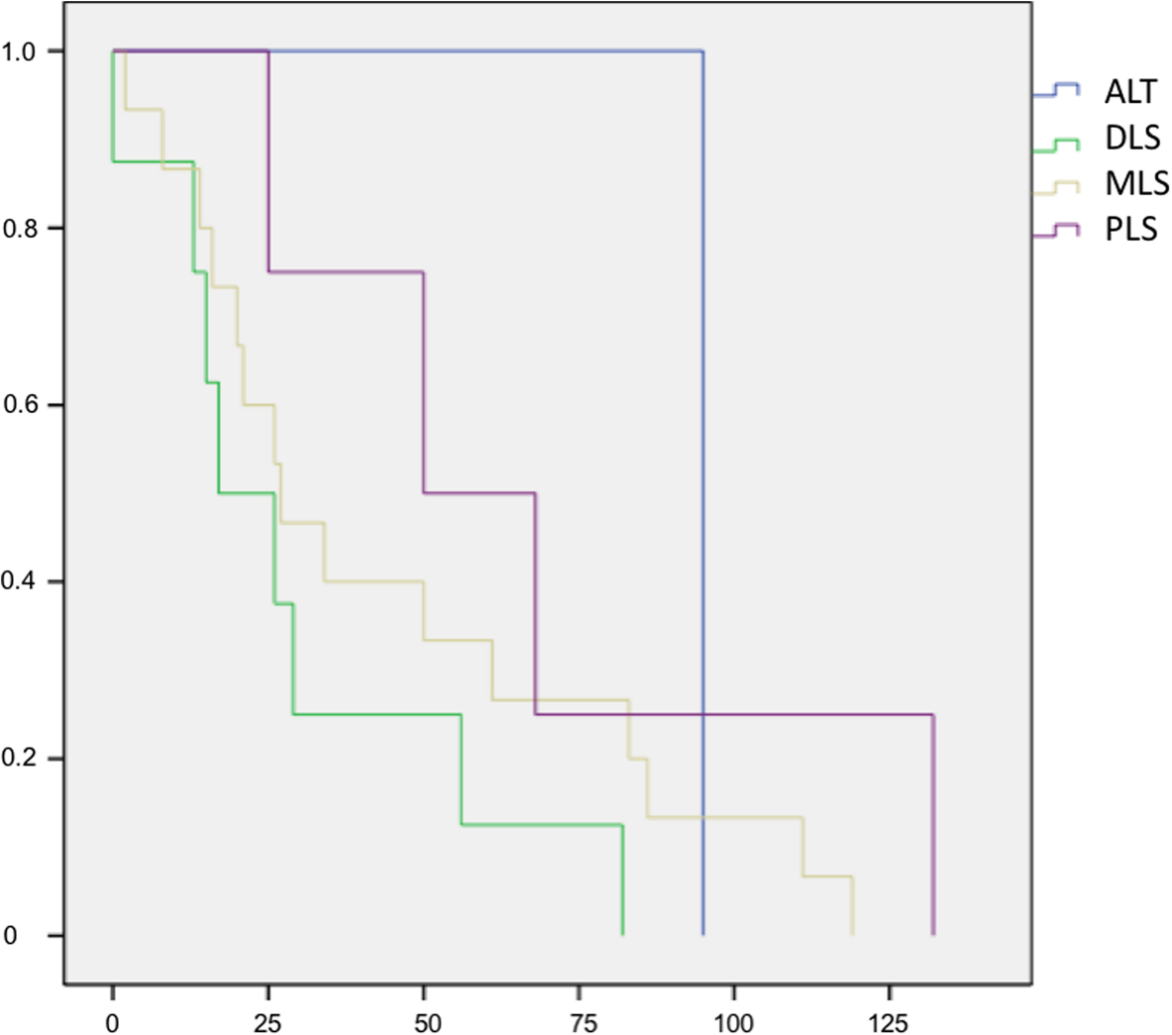
Kaplan-Meier Curve showing statistical analysis (x-axis: months, y-axis: probability) of survival without metastases in patients with metastases in months after initial diagnosis of different types of liposarcoma; *p*-value: *p* > 0.05. *blue line*: ALT (atypical lipomatous tumour), *green line*: DLS (dedifferentiated liposarcoma), *yellow line*: MLS (myxoid liposarcoma), *purple line*: PLS (pleomorphic liposarcoma)

**Fig. 3:**
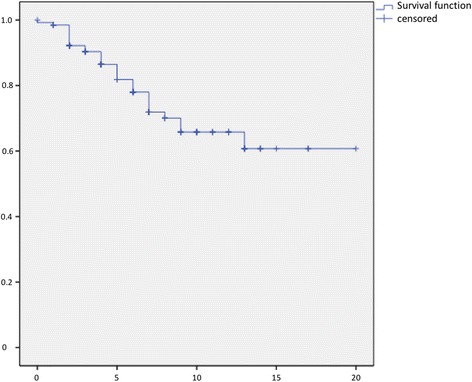
Kaplan-Meier Curve showing statistical analysis (x-axis: years, y-axis: probability) of overall survival in years after initial diagnosis of liposarcoma. *Blue line*: survival function

**Fig. 4:**
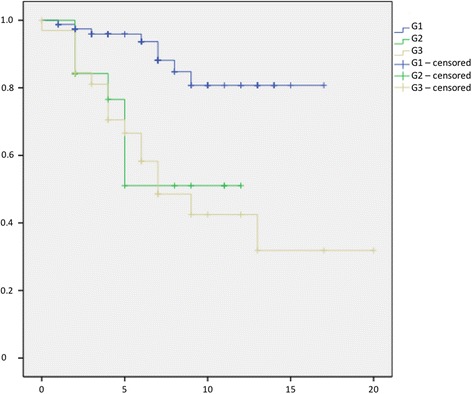
Kaplan-Meier Curve showing statistical analysis (x-axis: years, y-axis: probability) of survival in years after initial diagnosis correlated to grading of liposarcoma; *p*-value: *p* < 0.01. *blue line*: Grading G1, *green line*: Grading G2, *yellow line*: Grading G3

**Fig. 5:**
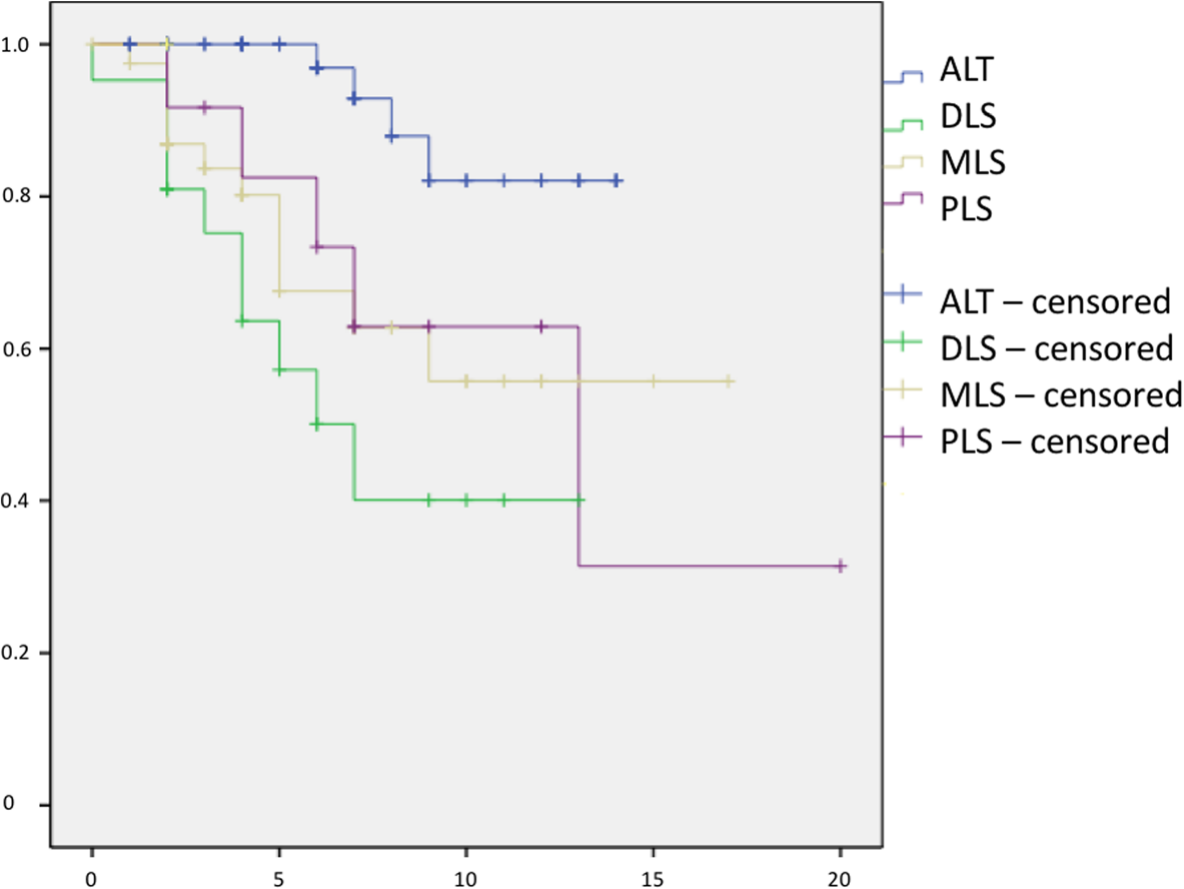
Kaplan-Meier Curve showing statistical analysis (x-axis: years, y-axis: probability) of survival in years after initial diagnosis correlated to histological subtyp of liposarcoma; *p*-value: *p* < 0.01. *blue line*: ALT (atypical lipomatous tumour), *green line*: DLS (dedifferentiated liposarcoma), *yellow line*: MLS (myxoid liposarcoma), *purple line*: PLS (pleomorphic liposarcoma)

**Fig. 6:**
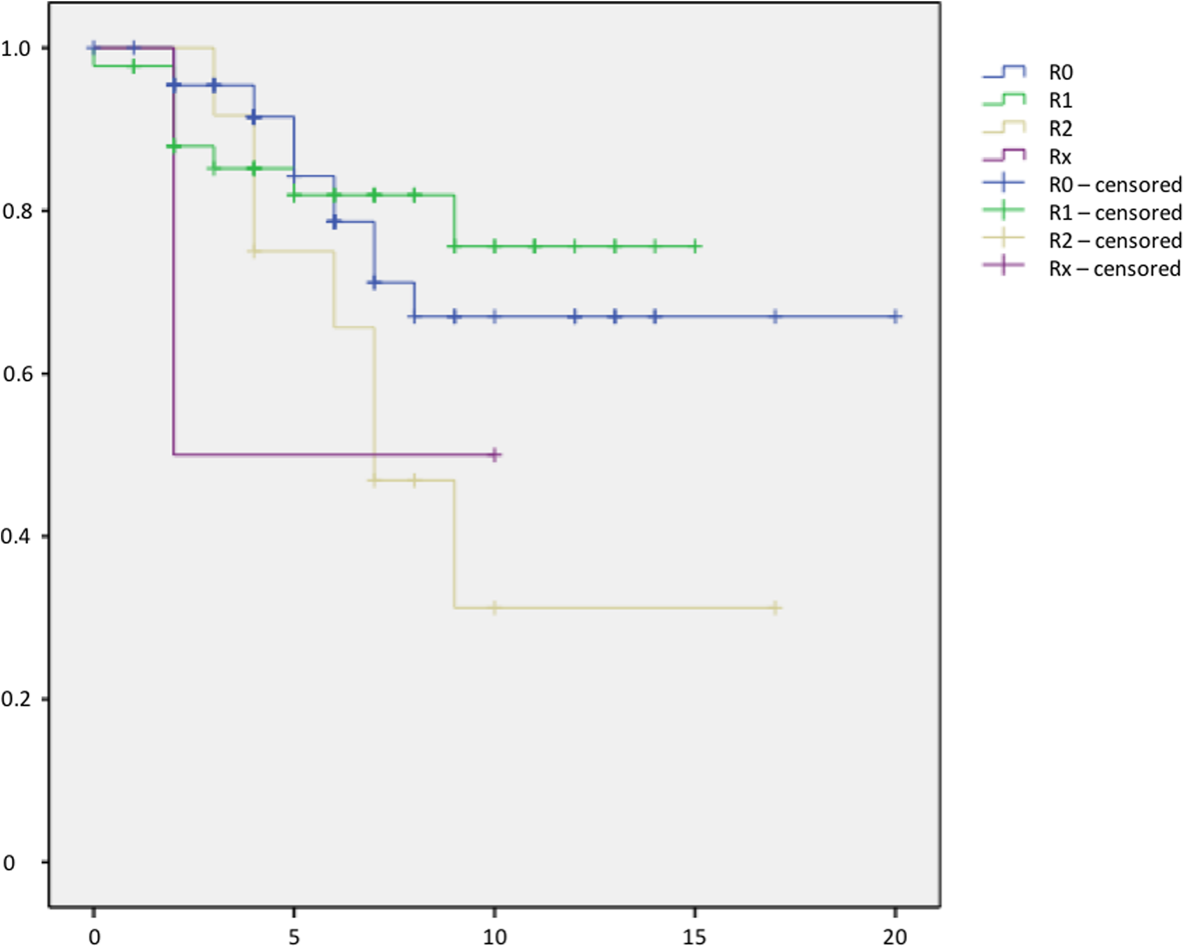
Kaplan-Meier Curve showing statistical analysis (x-axis: years, y-axis: probability) of survival in years after initial diagnosis correlated to resection margins of liposarcoma; *p*-value: *p* > 0.05. *blue line*: Resection margin R0, *green line*: Resection margin R1, *yellow line*: Resection margin R2, *purple line*: Resection margin Rx

**Fig. 7:**
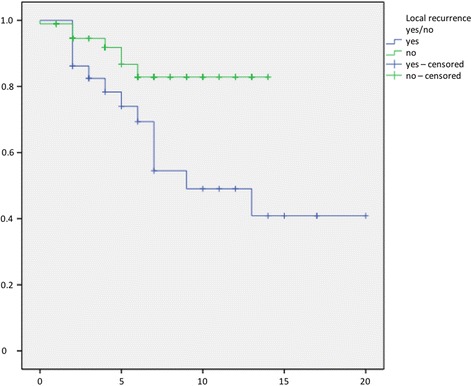
Kaplan-Meier Curve showing statistical analysis (x-axis: years, y-axis: probability) of survival in years after initial diagnosis correlated to local recurrence of liposarcoma; *p*-value: *p* < 0.01. *green line*: no recurrence of liposarcoma, *blue line*: recurrence of liposarcoma

**Fig. 8:**
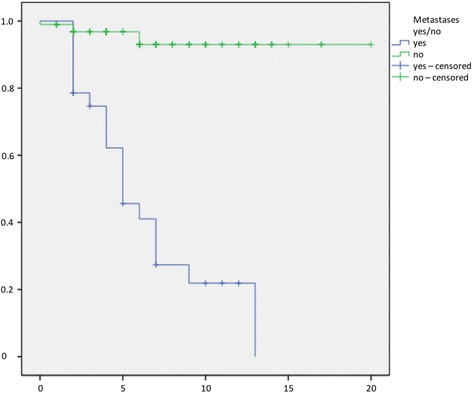
Kaplan-Meier Curve showing statistical analysis (x-axis: years, y-axis: probability) of survival in years after initial diagnosis correlated to metastases of liposarcoma; *p*-value: *p* < 0.01. *green line*: no metastases of liposarcoma, *blue line*: metastases of liposarcoma

The time to first local recurrence is illustrated in Fig. 1. There was a mean time to local recurrence for well-differentiated LS of 34 months (3–113), for well-differentiated LS of 26.9 (6–77), for myxoid LS of 59.1 (15–138), and 27 months (10–61) for dedifferentiated LS. We observed metastases in 22.6% (95%-CI 15.2–29.9) of all patients. Additionally, 46.4% (95%-CI 28.0–64.9) had G3 tumours, 39.3% (95%-CI 21.2–57.4) G2, and 14.3% (95%-CI 1.3–27.2) G1. Survival without metastases is illustrated in Fig. 2. Twenty-nine patients (21.8%, 95%-CI 14.8–28.8) died due to the diagnosis LS in our follow-up period. The survival rates depended on the grade, subtype, negative margin recrudescence, metastasis and tumour size (Figs. 3, 4, 5, 6, 7 and 8). Cox-regression and the log-rank test demonstrated significant single prognostic factors (existence of metastases) and subtype (dedifferentiated LS) on the survival rates as well as significant (*p* = 0.015) combined factors (grading and negative margins). Patients with G2 liposarcoma showed significantly worse results in the overall survival than patients with G1 liposarcoma (*p* = 0.004; Hazard Ratio 8.1; 95%-CI 1.9–34.1) as well as patients with G3 liposarcoma compared to patients with G1 liposarcoma (*p* = 0.034; Hazard Ratio 4.7; 95%-CI 1.1–19.7).

## Discussion

Liposarcoma accounts for approximately 20% of sarcoma in adults; therefore, it is the most frequently encountered malignant soft tissue tumour in clinical practice [11]. Its histological findings vary from well-differentiated and myxoid tumours to cellular, pleomorphic, and dedifferentiated neoplasms [12]. The histologic subgroups of LS have different natural clinical courses in terms of the clinical features and survival outcome [8]. However, current clinical practice is not optimized according to different histologic subtypes and clinical protocols, and it often does not reflect such a difference. In this study, we retrospectively analysed a single-centre cohort of patients with a history of LS and then evaluated the prognostic factors and their influence on mortality. The survival rates were highly dependent on the grading, LS subtype, negative margins after surgery, metastases and tumour size. Additionally, the existence of metastases and subtype were found to be major single prognostic factors affecting the survival rates. To the best of our knowledge, this is the most up-to-date report providing a comprehensive epidemiological and prognostic evaluation in a large population treated at a specialized centre.

In the present study, the average age of all patients was 55.1 years (range, 14–86). Patients with myxoid LS were significantly younger (average age: 50 years) than patients with well-differentiated tumours (average age: 62 years). This is in accordance with the largest series of malignant lipomatous tumours (910 patients), which was published by Dalal et al. [8]. In contrast to these findings, Kransdorf et al. reported slightly later occurrences for high-grade LS (>60 years) compared to well-differentiated tumours (50 years) [13].

The most common subtypes in the present study were well-differentiated and myxoid LS, which accounted for approximately 43.1% and 30.8%, respectively. Dedifferentiated (16.2%) and pleomorphic (9.2%) LS were much less common. The overall survival was well stratified by the histologic subtype: Well-differentiated LS had 5- and 10-year survival rates of 100% and 82.1% and dedifferentiated LS had survival rates of 57.2% and 40.1%. These survival rates are in accordance with the findings of Dalal et al. [8], but they only partially agreed with the large series by Fletscher et al., for which the survival for dedifferentiated LS was 70% and pleomorphic LS was 55–65% [14].

In terms of the tumour burden, patients with a tumour diameter less than 5 cm had a prolonged overall survival compared to those with a diameter greater than 5 cm. The survival rates were 80% and 67.3% for patients with a tumour diameter smaller and greater than 5 cm, respectively. Because only 5 patients with a tumour size smaller than 5 cm were included in the present study (versus 111 patients with a size >5 cm), a significant difference between the overall survival rates was not detectable.

Regarding the primary sites, nearly three-fourths of tumours were located in the lower extremities. This finding only partially reflects prior observations in previous studies of lower incidences of LS of the lower extremities, which were higher for retroperitoneal localizations [15–17]. In addition, the present study did not demonstrate that the extremity sites had a significantly favourable survival compared with retroperitoneal sites, as stated before [8]. However, we also analysed the epi- or subfascial sites independently from the body region. Epifascial localization showed the most favourable prognosis (5- and 10-year survival of 84.6%), while subfascial tumours demonstrated a reduced 10-year survival rate of 64.21% in the present study. There was no significant difference in those two groups because of the major differences in the number of patients (13 vs. 112). Several authors found similar distribution patterns of LS regarding epi- and subfascial localization, but these could not be correlated with the survival rate [6, 10, 15, 18].

Several authors described the resection margin as one of the most important factors affecting survival [5, 13, 16]. We analysed resection margins by gross and microscopic examinations and then defined a clear margin when there was no tumour at least 1 mm or more from the edge of the inked specimen. Clear margins were detected in 53.6%; all others had either microscopically or macroscopically positive margins. The survival rates of patients with clear margins were 100%, 84.3% and 67% after 1, 5, and 10 years. On the other hand, if positive margins were only microscopically detected, the corresponding survival rates were 97.7%, 81.9%, and 75.6%. Additionally, if a macroscopically tumour was left in place, the respective survival rates were 100%, 75.0% and 31.3%. In the present study, no statistically significant relationship was found between the margins and survival. One possible explanation for the higher 10-year survival rate after microscopically positive margins might be that five patients had clear margins after a second surgery, and some of these patients were treated with radiotherapy. Nevertheless, this is not the first study reporting that margins do not necessarily correlate with survival [19]. In contrast to our findings, several authors found the highest survival in patients with clear margins and the lowest survival in patients with macroscopic tumour residuals or patients considered for amputation due to extensive tumour growth [6–8, 20].

In terms of the histologic status, 47.6% and 36.4% of patients with dedifferentiated and pleomorphic LS developed local recurrence in the present study. In terms of localization, retroperitoneal LS showed local recurrence in 80% of cases, although no tumours were initially resected with free margins. Regarding the time of local recurrence, nearly 50% of recurrences occurred within the first two years after primary surgery. Another 30% developed local recurrence between the third and fifth years following surgery, and approximately 20% developed recurrence from the fifth year on. The mean recurrence-free survival was 37.8 months in our study. This finding almost perfectly reflects the findings of the largest series of LS from Dalal et al. These authors found a recurrence free survival time of 35 months [8]. Local recurrence in the present study was associated with decreased survival. Patients with local recurrence showed 10- and 5-year survival rates of 86.2% and 49.0%, which was significantly lower than the survival of patients without recurrence (86.7% and 82.9% at 10 and 5 years). Local recurrence is reported to vary between 13 and 97% and is highly dependent on the histologic type [9, 18, 21, 22].

Twenty-eight patients (22.4%) developed distant metastases during the study period. Metastases were localized in the bone, lungs, soft tissues and lymph nodes. The median time to the development of the first metastasis was 27 months, and only a few cases had metastases ten years from surgery. In the literature, the development of metastases is reported to vary between 30 and 50%; this is similar to the risk of local recurrence, which strongly depends on the histologic subtype and grading [6, 9, 10, 19]. In our series, we found one patient with well-differentiated LS who developed a distant metastasis at 95 months after surgery. This has not previously been described. In this particular case, the patient suffered from a retroperitoneal tumour that was not resected with clear margins. One possible explanation for this late metastatic process could be that this tumour underwent dedifferentiation over time. However, from a prognostic point of view, we found a clear correlation between metastasis and survival. Patients who developed distant metastasis after surgery showed 5- and 10-year survival rates of 45.6% and 21.9%, which were 96.8% and 93.0% for patients without metastases. These rates are reported in similar magnitude as the results reported by several authors [8, 10, 11].

This study has several limitations that merit discussion. First, the retrospective study design is subject to recall and selection bias. The number of patients in the groups sometimes differed, making comparison very difficult. Nevertheless, due to the rare incidence of this tumour, our series is comparable to previously published studies. Second, this study lacks a control group. Therefore, we cannot directly compare the treatment results with other types of regimens. Third, a minimum follow-up period of 5 years was not possible for all patients. This might influence the definitive evaluation of the outcomes for this tumour type. Studies with longer-term follow-up are necessary because the survival rates are easily underestimated in a shorter time period.

## Conclusion

Grading, the liposarcoma subtype, negative margins after surgery, metastases and tumour size are independently associated with disease-specific survival, and patients with local recurrence had lower survival rates. We hope our investigation may facilitate further prospective study and clinical decision-making in liposarcoma.

## Abbreviation

LS: Liposarcoma
